# Muscle Synergies: Use and Validation in Clinics, Robotics, and Sports

**DOI:** 10.1155/2018/6345256

**Published:** 2018-12-20

**Authors:** Stefano Rossi, Panagiotis K. Artemiadis, Jinsook Roh, Valentina Agostini

**Affiliations:** ^1^Department of Economics, Engineering, Society and Business Organization, University of Tuscia, Viterbo, Italy; ^2^Department of Mechanical and Aerospace Engineering, Arizona State University, Tempe, AZ, USA; ^3^Department of Biomedical Engineering, University of Houston, Houston, TX, USA; ^4^Department of Kinesiology, Temple University, Philadelphia, PA, USA; ^5^Department of Physical Medicine and Rehabilitation, Northwestern University, Chicago, IL, USA; ^6^Department of Electronics and Telecommunications, Politecnico di Torino, Torino, Italy

Since understanding how the human brain generates neural commands to control muscles during motor tasks still remains an untapped question, great interest is shown in the validation and application of muscle synergies among research groups focused on the electromyography (EMG). In the last decades, the factorization of the EMG signals by means of muscle synergies has been proposed to understand the neurophysiological mechanisms related to the central nervous system ability in reducing the dimensionality of muscle control. For this reason, we planned a special issue on validation and application of the muscle synergy theory to discuss the methodological issues and to propose novel applications in clinics, robotics, and sports. The special issue achieved success among researchers as demonstrated by the large amount of submitted papers and the scientific impact of the published ones.

The special issue is composed of twelve manuscripts. Three systematic reviews are included: (i) the first one is focused on the meaning of the muscle synergy theory to understand its applicability as a neurorehabilitation tool (Singh et al.); (ii) the second one is useful to understand the applications of muscle synergies in the investigation of muscle coordination during walking of poststroke patients (Seamon et al.); and (iii) the third one offers a complete overview on the tangible applications of muscle synergies in clinics, robotics, and sports (Taborri et al.).

As concerns clinics, the effects of upper limb weakness and task failure, which is the inability to maintain a certain level of force during a task, on the muscle synergies are evaluated by Roh et al. and Castronovo et al., respectively. As regards robotics, the feasibility to use a muscle synergy approach to implement the control system of an upper limb exoskeleton is presented by Chiavenna et al. Moving to the sports, two papers are focused on understanding the muscle synergy organization during the execution of specific technical actions of the badminton (Matsunaga et al. and Barnamehei et al.), one paper shows the muscle synergy structure involved in stability exercises of rhythmic gymnastics (Rutkowska-Kucharska et al.), while the motor control underlying the throwing movement is studied by Cruz-Ruiz et al. Finally, two papers investigate some fundamental methodological issues; in particular concerning the influence of initialization techniques for the application of non-negative matrix factorization (Soomro et al.) and the reliability and repeatability of the methodology for extracting muscle synergies during daily life activities (Taborri et al.).

We hope that this special issue can represent an important step to strengthen the use of muscle synergies to explain how the human brain organizes the muscle activation both in clinics and robotics, as well as in sports applications.

